# Green Extraction and Characterization of Collagen from Different Fish Scales and Cell Culture-Based Bioactive Evaluation of Its Combinations with *Zingiber officinale*

**DOI:** 10.3390/polym18151799

**Published:** 2026-07-23

**Authors:** Ayşe Kara, Hatice Onay, Elif Arslan, Züleyha Akpınar Emanet, Arzu Düdükçü, Hasan Türkez

**Affiliations:** 1 Faculty of Fisheries, Recep Tayyip Erdogan University, Rize 53100, Turkey; ayse.kara1@erdogan.edu.tr (A.K.); zuleyha_akpinar17@erdogan.edu.tr (Z.A.E.); arzu_dudukcu23@erdogan.edu.tr (A.D.); 2 Department of Medical Biology, Faculty of Medicine, Atatürk University, Erzurum 25240, Turkey; elif.arslan252@ogr.atauni.edu.tr (E.A.); hturkez@atauni.edu.tr (H.T.)

**Keywords:** red mullet, NADES, *Zingiber officinale*

## Abstract

Fish processing by-products represent a valuable and sustainable source of collagen for biomedical and functional applications. In the present study, collagen was extracted from the scales of red mullet (*Mullus barbatus*), gilthead sea bream (*Sparus aurata*), and European sea bass (*Dicentrarchus labrax*) using a green extraction approach based on a natural deep eutectic solvent (NADES) system composed of citric acid, xylitol, and water. The extracted collagens were comprehensively characterized by SDS-PAGE, Fourier transform infrared spectroscopy (FTIR), scanning electron microscopy (SEM), and X-ray diffraction (XRD) analyses. The results confirmed the presence of Type I collagen and demonstrated that the characteristic triple-helical structure and molecular organization were preserved throughout the extraction process. To preliminarily evaluate their potential as matrices for incorporating bioactive compounds, collagen matrices were combined with *Zingiber officinale* (ginger) extract at different ratios. The resulting combinations were evaluated for antioxidant, antimicrobial, and cytocompatibility properties. Antioxidant activity increased significantly with increasing ginger concentration, with the highest radical scavenging activity observed in combinations containing the greatest proportion of ginger extract. Similarly, antimicrobial activity against Escherichia coli and Staphylococcus aureus was enhanced by ginger incorporation, with stronger inhibition observed against the Gram-positive bacterium. Cytocompatibility studies performed on human dermal fibroblast (HDFa) cells revealed that selected collagen–ginger combinations maintained high cell viability and did not induce substantial membrane damage, nuclear abnormalities, apoptosis, or necrosis at appropriate concentrations. Overall, the findings demonstrate that fish scale-derived collagen obtained through a sustainable NADES-based extraction process possesses favorable structural and biological properties and may serve as a potential carrier matrix for plant-derived bioactive compounds. These findings provide preliminary evidence supporting the future development of environmentally friendly functional biomaterials with prospective applications in pharmaceutical, biomedical, and tissue engineering fields.

## 1. Introduction

Collagen, recognized for its triple-helical structure, is considered one of the most fundamental structural proteins in vertebrates. Due to its high biocompatibility, biodegradability, and low antigenicity, it has been safely utilized for many years in the food, pharmaceutical, biomedical, and cosmetic industries [1]. In conventional collagen production, the skin and bone tissues of cattle and pigs are primarily used as raw materials. However, outbreaks such as bovine spongiform encephalopathy and foot-and-mouth disease, particularly in cattle, have significantly undermined consumer confidence in collagen and collagen-based products derived from these animals [2]. These concerns have directed both researchers and industry toward alternative collagen sources, and collagen derived from aquatic organisms has attracted increasing attention in recent years. By-products generated during fish processing, such as skin, scales, bones, and swim bladders, present considerable potential for collagen production [3]. The rapid expansion of the fish processing industry inevitably results in the generation of larger quantities of by-products. Therefore, the use of fish processing by-products as an alternative raw material for collagen production stands out as a sustainable and strategic approach, both by providing economic added value to the fisheries sector and by reducing environmental burdens through recycling. Deep eutectic solvents are defined as mixtures of two or more components that exhibit a significant decrease in melting point when combined in specific ratios, remaining in liquid form at room temperature. Formed through strong interactions between a hydrogen bond acceptor and a hydrogen bond donor, these solvents provide an environmentally friendly and sustainable alternative to conventional industrial solvents [4]. Generally, the interaction of the complex-forming component, which acts as a hydrogen bond donor, with halide anions weakens the anion–cation interactions, thereby lowering the melting point of the mixture far below that of the individual components. Interest in these solvents increases further when they are composed of natural constituents such as urea, oxalic acid, ethylene glycol, and choline chloride. Owing to these properties, they provide a highly suitable medium for the extraction of volatile aromatic and phenolic compounds, metals, and collagen proteins [5]. In addition, their low cost, the possibility of numerous different combinations, biodegradability, and low toxicity make deep eutectic solvents a promising option for current research and industrial applications [6]. Furthermore, the combination of collagen and NADESs has been reported to enhance the natural wound-healing properties of collagen while also increasing the potential antibacterial effects of NADESs [7]. In addition to their ability to dissolve collagen, NADESs can preserve its structural integrity, allowing these solvent systems to be considered as potential auxiliary components in collagen-based biomaterials and biomedical products [7].

The main objective of this study was to contribute to recycling processes through the valorization of fish processing waste within the framework of sustainable fisheries. In this context, the scales of gilthead seabream (*Sparus aurata*) and European seabass (*Dicentrarchus labrax*), which are widely cultured species, as well as red mullet (*Mullus barbatus*), an economically important wild-caught species, were used as raw materials. The study was based on the hypothesis that high-quality collagen with preserved structural integrity can be obtained from these fish scales using environmentally friendly and sustainable extraction methods. Therefore, the extracted collagens were subjected to comprehensive structural and physicochemical characterization. Furthermore, the potential of the obtained collagens as candidate carrier matrices for natural bioactive compounds was preliminarily evaluated by physically combining them with *Zingiber officinale* (ginger) extract. To assess the ability of the collagen matrix to incorporate and protect ginger-derived bioactive compounds, antioxidant and antimicrobial activities were determined. In addition, biocompatibility studies were conducted to explore the suitability of these systems for future biomedical and functional biomaterial applications.

## 2. Materials and Methods

In this study, two aquaculture fish species, European seabass and gilthead seabream, together with red mullet obtained through commercial fishing, were supplied from commercial fishery companies. The fish samples brought to the laboratory were thoroughly washed with plenty of water to remove surface impurities and residues. Following the washing process, the scales were separated from the fish using scalpels and similar surgical instruments. The collected scales were transferred into sterile plastic bags and stored at −20 °C until further analyses. Fresh ginger (*Zingiber officinale*) rhizomes were purchased from a local commercial market (Migros, Rize, Türkiye) under the Verita^®^ brand, which supplies GLOBALG.A.P.-certified products, and were used for extract preparation.

### 2.1. Preparation of the Natural Deep Eutectic Solvent (NADES) and Collagen Extraction

The NADES containing citric acid, xylitol, and water (molar ratio 1:1:10) was prepared based on the method described by [7] with slight modifications. The three-component mixture was transferred into a round-bottom flask and stirred in a water bath at 50 ± 5 °C for approximately 2 h until a homogeneous and transparent liquid was obtained. Frozen fish scales were freeze-dried for 48 h and subsequently ground into powder form. The obtained scale powder was mixed with NADES at a ratio of 1:10 (*w*:*v*) and subjected to extraction at 40 °C under continuous stirring for 1 h. At the end of the extraction period, the mixture was centrifuged at 4500 rpm for 20 min. The supernatant was dialyzed against fresh distilled water for 72 h, with the water being replaced every 12 h. Following dialysis, the extract was freeze-dried for 24 h and stored at room temperature for further analyses. Fresh ginger samples were dried at 50 °C for 24 h and then ground into a fine powder using a household grinder. The extraction procedure was carried out by mixing 10 g of ginger powder with 100 mL of 70% (*v*/*v*) ethanol at a solid-to-solvent ratio of 1:10 (*w*/*v*). The mixture was extracted in an ultrasonic water bath (40 kHz) at 30–35 °C for 30 min. After extraction, the mixture was first filtered through Whatman No. 1 filter paper to remove coarse particles. The resulting filtrate was subsequently passed through a 0.20 µm membrane filter to ensure microbiological purity. The extract was stored in dark-colored bottles at 4 °C until use.

### 2.2. Collagen Characterization

#### 2.2.1. Sodium Dodecyl Sulfate–Polyacrylamide Gel Electrophoresis (SDS-PAGE)

SDS-PAGE analysis was performed based on the protocol described by [8] with minor modifications. Collagen samples were first dissolved in 0.1 M acetic acid. The dissolved samples were mixed at ratios of 1:1 and 1:2 (*v*/*v*) with a sample buffer containing 30% (*v*/*v*) glycerol and 5% (*w*/*v*) SDS prepared in 0.5 M Tris–HCl buffer (pH 6.8). In addition, 10% (*v*/*v*) mercaptoethanol and 0.04% (*w*/*v*) bromophenol blue were added to the mixture. Following electrophoresis, the gel was stained with Coomassie Brilliant Blue G-250 to visualize the protein bands. The molecular weights of the proteins were estimated using high-molecular-weight protein markers. Gel images were analyzed using a gel documentation system (G:BOX Chemi HR16, Syngene, Cambridge, MA, USA) through the GeneSys software.

#### 2.2.2. FTIR (Fourier Transform Infrared Spectroscopy)

FTIR analysis of the extracted lyophilized collagen was carried out using a Perkin Elmer Spectrum 100 FTIR spectrophotometer (PerkinElmer, Shelton, CT, USA). The interpretation of the obtained spectral peaks was based on the reference findings reported by [9]. For the analysis, 5 mg of lyophilized collagen sample was directly placed onto the crystal cell of the instrument. The spectra of all collagen samples were recorded against a background spectrum within the wavenumber range of 4000–400 cm^−1^, at a spectral resolution of 2 cm^−1^ with 32 scans.

#### 2.2.3. SEM (Scanning Electron Microscopy)

Prior to analysis, lyophilized collagen samples were sputter-coated with gold for 5 min using a JEOL JFC-1200 (Tokyo Rikakikai Co., Ltd., Tokyo, Japan) fine coater. After the coating process, the surface morphology and structural properties of the collagen samples were examined using a scanning electron microscope (SEM; Phillips XL 30 SFEG, Thermo Fisher Scientific, Waltham, MA, USA) [10] (Raub et al., 2008).

#### 2.2.4. X-Ray Diffraction Analysis (XRD)

The crystalline structure of the lyophilized collagen was analyzed using a Rigaku D-Max RNIT 2200 X-ray diffractometer (Rigaku Corporation, Tokyo, Japan). Measurements were performed using Cu Kα radiation generated from a rotating anode generator under operating conditions of 45 kV and 30 mA. Diffraction patterns were recorded within a 2θ range of 4–60°, with a scanning step of 0.02° and a scanning rate of 2°/min.

### 2.3. Preparation of Ginger Extract

Fresh ginger samples were dried at 50 °C for 24 h and then ground into a fine powder using a household grinder. The extraction procedure was performed by mixing 10 g of ginger powder with 100 mL of 70% (*v*/*v*) ethanol at a solid-to-solvent ratio of 1:10 (*w*/*v*). The mixture was extracted in an ultrasonic water bath (Weightlab WF-Ud4) at 30–35 °C for 30 min, 40 kHz. After extraction, the mixture was first filtered through Whatman No. 1 filter paper to remove coarse particles. The obtained filtrate was subsequently passed through a 0.20 µm membrane filter to ensure microbiological purity. The extract was stored in dark-colored bottles at 4 °C until use.

### 2.4. Preparation of Collagen Solution

Fish collagens obtained from gilthead seabream (*Sparus aurata*), red mullet (*Mullus barbatus*), and European seabass (*Dicentrarchus labrax*) were prepared in pure water at a concentration of 4% (*w*/*v*). For this purpose, 4 g of collagen was added to a defined volume of pure water and stirred on a magnetic stirrer at 45 °C until completely dissolved. After cooling to room temperature, the final volume was adjusted to 100 mL with pure water.

### 2.5. Preparation of Collagen–Ginger-Based Combinations

Stock collagen solutions were prepared at a concentration of 4% (*w*/*v*), while ginger extract stock solutions were prepared at a concentration of 10% (*v*/*v*). Collagen–ginger combinations were produced by mixing collagen solutions derived from red mullet (*Mullus barbatus*), European seabass (*Dicentrarchus labrax*), and gilthead seabream (*Sparus aurata*) with the ginger extract stock solution at collagen-to-extract volumetric ratios of 4:1, 3:2, and 2:3, generating the experimental groups M1–M3, S1–S3, and D1–D3, respectively. Combinations containing only collagen stock solution (Mc, Sc, and Dc) were used as controls (Table 1). The total volume of each combination was maintained constant, and the mixtures were homogenized by vortex mixing for approximately 10 min. The pH of each combination was subsequently measured and adjusted to 5.8 by the dropwise addition of 0.1 M NaOH. Following pH adjustment, the combinations were allowed to equilibrate at room temperature for 15 min prior to further analyses. To preserve the integrity of the collagen–ginger bioactive complexes and minimize the loss of associated bioactive compounds, the final combinations were not subjected to membrane filtration. All samples were stored at 4 °C until further characterization and biological analyses.

### 2.6. 2,2-Diphenyl-1-picrylhydrazyl Hydrate (DPPH) Radical Scavenging Activity

The antioxidant activity of the samples was determined using the DPPH (2,2-Diphenyl-1-picrylhydrazyl hydrate) radical scavenging capacity method [11]. The DPPH stock solution was prepared by dissolving DPPH in 100 mL of methanol and stored at −20 °C until use. The working solution was prepared by mixing 10 mL of the stock solution with 45 mL of methanol and adjusting the absorbance to 1.1 ± 0.02 at 515 nm against a methanol blank sample. For the analysis, 100 µL of sample solution was mixed with 3900 µL of DPPH solution and incubated at room temperature in the dark for 45 min. To establish the standard calibration curve, 100 µL aliquots of Trolox solutions prepared at concentrations of 100, 200, 400, 600, 800, and 1000 µM were mixed with 3.9 mL of DPPH working solution. At the end of the reaction period, absorbance values were measured at 515 nm using a UV–Vis spectrophotometer. The results were expressed as percentage inhibition.

The free radical scavenging activity was calculated using the following equation:

$$\%Inhibition\;=(\frac{A_{blank}\;-\;A_{sample}}{A_{blank}})\;\times 100$$

where $A_{blank}$: absorbance value of the blank sample, $A_{sample}$: absorbance value of the sample.

### 2.7. Antimicrobial Activity

Antimicrobial activity was determined according to the agar-well diffusion method. Sample solutions (30 μL) were added into wells (6 mm) cut into tryptic soy agar (TSA) plates previously inoculated with each bacterial strain (*Escherichia coli* and *Staphylococcus aureus*). For the control group, 30 μL of PBS (pH 7.2) was added into empty wells without sample. The plates were incubated at 37 °C for 24 h. After incubation, the diameters of the inhibition zones were measured. The experiments were performed in triplicate, and the inhibition zone diameters were recorded [12].

### 2.8. MTT Cell Viability Assay

Cell viability was evaluated using the MTT colorimetric assay to determine the cytocompatibility of D3, M3, and S3 combinations in human dermal fibroblast cells (HDFas). Human adult dermal fibroblasts (HDFas; ATCC^®^ PCS-201-012™) were obtained from ATCC (Manassas, VA, USA) and cultured according to the supplier’s instructions. Since commercially available human primary cells were used, no additional ethical approval was required. Briefly, HDFa cells were seeded into 96-well cell culture plates at a density of approximately 1 × 10^4^ cells/well in complete culture medium and incubated at 37 °C in a humidified atmosphere containing 5% CO_2_ for 24 h to allow cell attachment. After incubation, the culture medium was removed, and cells were treated with serial concentrations of each combination, including 1.56%, 0.78%, 0.39%, 0.20%, 0.10%, 0.05%, 0.02%, and 0.01%, for 24 h. Untreated cells were used as the negative control. Following treatment, the medium was aspirated, and MTT reagent was added to each well according to the manufacturer’s instructions using an MTT Cell Proliferation Assay Kit (Abcam, Cambridge, UK). The cells were incubated for approximately 3–4 h at 37 °C to allow viable cells to reduce MTT into insoluble formazan crystals. After incubation, the formazan crystals were dissolved using the kit solubilization solution, and absorbance was measured at 570 nm using a microplate reader. Cell culture reagents, including Dulbecco’s Modified Eagle Medium (DMEM), fetal bovine serum (FBS), penicillin–streptomycin, phosphate-buffered saline (PBS), and trypsin–EDTA, were obtained from Sigma-Aldrich (St. Louis, MO, USA). Cell viability was calculated as a percentage relative to the negative control using the following equation:

$$Cell\;viability\;\left(\%\right)=\frac{Absorbance\;of\;treated\;cells}{Absorbance\;of\;negative\;control\;cells}\times 100$$

All experiments were performed in triplicate, and the results were expressed as mean ± standard deviation (SD). Statistical comparisons between treated groups and the negative control were performed using one-way analysis of variance followed by an appropriate post hoc test, with *p* < 0.05 considered statistically significant.

### 2.9. FDA/PI Fluorescent Cell Viability Assay

Fluorescein diacetate/propidium iodide (FDA/PI) staining was performed to qualitatively evaluate membrane integrity and cell viability in HDFa cells following exposure to selected combination concentrations. Based on the MTT cell viability results, cells were treated with D3 at 0.20%, M3 at 0.05%, and S3 at 0.05% for 24 h. Untreated cells were used as the negative control. Briefly, HDFa cells were seeded into 24-well plates at a density of 5 × 10^4^ cells/well and incubated at 37 °C in a humidified atmosphere containing 5% CO_2_ for 24 h to allow cell attachment. After treatment, the culture medium was removed, and the cells were gently washed with phosphate-buffered saline (PBS). The cells were then stained with fluorescein diacetate (FDA; Sigma-Aldrich, St. Louis, MO, USA) and propidium iodide (PI; Sigma-Aldrich, St. Louis, MO, USA) prepared in PBS. FDA was used to identify viable cells with intact membrane integrity and intracellular esterase activity, whereas PI was used to detect non-viable cells with compromised plasma membranes. Following staining, the cells were incubated in the dark at room temperature for the appropriate staining period. The staining solution was then removed, and the cells were immediately examined using an inverted fluorescence microscope. FDA-positive viable cells were visualized as green fluorescent cells, while PI-positive non-viable cells were visualized as red fluorescent cells. Representative micrographs were captured from each experimental group.

### 2.10. Giemsa Staining for Nuclear Morphology Analysis

Giemsa staining was performed to evaluate nuclear integrity and combination-induced nuclear abnormalities in human dermal fibroblast cells (HDFas) following exposure to the selected concentrations of D3, M3, and S3. Based on the MTT and FDA/PI cell viability findings, HDFa cells were treated with D3 at 0.20%, M3 at 0.05%, and S3 at 0.05% for 24 h. Untreated cells were used as the negative control. Briefly, HDFa cells were seeded into 24-well plates and incubated at 37 °C in a humidified atmosphere containing 5% CO_2_ until adequate attachment was achieved. After 24 h of treatment, the culture medium was removed, and the cells were gently washed with phosphate-buffered saline (PBS). Cells were then fixed using methanol (Sigma-Aldrich, St. Louis, MO, USA) for approximately 10 min at room temperature. Following fixation, the cells were stained with Giemsa solution (Sigma-Aldrich, St. Louis, MO, USA) prepared according to the manufacturer’s recommendations. After staining, excess dye was removed, and the wells were carefully washed with distilled water and allowed to air-dry. Stained cells were examined under a light microscope to assess cellular and nuclear morphology. Nuclear abnormalities, including micronuclei, lobbed nuclei, and notched nuclei, were recorded for each experimental group. For quantitative evaluation, 1000 cells per group were examined, and the frequency of nuclear abnormalities was calculated and expressed as mean nuclear abnormalities/1000 cells ± SD.

### 2.11. Annexin V-FITC/PI Flow Cytometry Analysis

Apoptotic and necrotic cell death mechanisms were evaluated using Annexin V-FITC/PI flow cytometry analysis in human dermal fibroblast cells (HDFas). Based on the preliminary MTT cell viability results, the selected combination concentrations were used for further analysis: D3 at 0.20%, M3 at 0.05%, and S3 at 0.05%. Untreated cells were included as the negative control. Cells were exposed to the combinations for 24 h under standard culture conditions at 37 °C in a humidified atmosphere containing 5% CO_2_. Briefly, HDFa cells were seeded into 6-well plates and allowed to attach overnight. After treatment, both floating and adherent cells were collected to avoid the loss of detached apoptotic or necrotic cells. Adherent cells were detached using trypsin–EDTA, combined with the corresponding culture medium, and centrifuged at approximately 300× *g* for 5 min. The cell pellets were washed with cold phosphate-buffered saline (PBS) and resuspended in binding buffer. Cell death analysis was performed using an Annexin V-FITC/Propidium Iodide Apoptosis Detection Kit (Sigma-Aldrich, St. Louis, MO, USA) according to the manufacturer’s instructions. The cell suspension was incubated with Annexin V-FITC and propidium iodide (PI) for approximately 15 min at room temperature in the dark. After incubation, samples were immediately analyzed by flow cytometry. Annexin V-FITC fluorescence was detected in the FL1 channel, while PI fluorescence was detected in the FL2 channel. Cell populations were classified according to their Annexin V-FITC/PI staining pattern as follows: Annexin V−/PI− cells were considered viable, Annexin V+/PI− cells were considered early apoptotic, Annexin V+/PI+ cells were considered late apoptotic/secondary necrotic, and Annexin V−/PI+ cells were considered necrotic. Data were expressed as the percentage of cells in each quadrant.

## 3. Results

### 3.1. Collagen Characterization

#### 3.1.1. SDS-PAGE (Sodium Dodecyl Sulfate–Polyacrylamide Gel Electrophoresis)

The protein patterns of NADES-dissolved collagen obtained from fish scales were analyzed using SDS-PAGE analysis (Figure 1). It was determined that the electrophoretic profiles of collagen exhibited three distinct bands. Among these bands, the first one, corresponding to molecular weights of 125–130 kDa (α1–α2), was observed to have a stronger staining intensity compared to the others. In addition, the formation of another band structure with a higher molecular weight (above 170 kDa), corresponding to the β component, was also observed.

#### 3.1.2. FTIR (Fourier Transform Infrared Spectroscopy)

The characteristic peak positions of the FTIR spectra are presented in Figure 2 and Table 2. The analytical results revealed the presence of the main amide bands specific to collagen, namely Amide I, Amide II, and Amide III, in all three species, indicating that the structural integrity was preserved. In addition, the Amide A band corresponding to N–H stretching vibrations was observed at approximately 3400 cm^−1^ in all samples. It has been reported in the literature that FTIR spectra of commercial collagen typically exhibit Amide A at approximately 3300 cm^−1^, Amide B at 2930 cm^−1^, Amide I at 1650 cm^−1^, Amide II at 1540 cm^−1^, and Amide III at 1235 cm^−1^. These characteristic bands confirm that the samples retained their protein structure and that the triple-helix conformation specific to collagen was preserved.

#### 3.1.3. SEM (Scanning Electron Microscopy)

Figure 3 presents the microstructural properties of collagen obtained from three different fish scales using the NADES method. As a result of SEM analyses, it was determined that the collagen samples did not exhibit significant impurities or salt residues associated with the extraction process and showed a partially smooth and homogeneous surface morphology. However, a complex structural organization composed of slightly wrinkled layers with irregular edges was observed. The slightly wrinkled and porous morphology observed on the surface of the samples is thought to have formed due to dehydration occurring during the freeze-drying process [13].

#### 3.1.4. X-Ray Spectrum

The X-ray diffraction (XRD) spectra of collagens obtained from three different fish scales using the NADES method were analyzed, and two characteristic diffraction peaks were identified in each sample (Figure 4). The diffraction angles (2θ) were determined to be approximately 8.2° and 20.48° for red mullet, 8.2° and 22.36° for gilthead seabream, and 7.92° and 22.54° for European seabass. These characteristic peaks indicate that the ordered molecular organization specific to the fibrillar structure of collagen was preserved.

Collagen yield was calculated according to the method described by [14]. The collagen yields obtained from the three fish species were determined as 1.32 g/100 g for gilthead seabream (*Sparus aurata*), 1.30 g/100 g for European seabass (*Dicentrarchus labrax*), and 1.11 g/100 g for red mullet (*Mullus barbatus*), based on 100 g of fish scales. In addition, the pH of the extracted collagen samples was determined according to the method described by [15]. The pH values of the collagen extracted from the three fish species were found to be [5.97 pH] for gilthead seabream (*Sparus aurata*), [5.99 pH] for seabass (*Dicentrarchus labrax*), and [5.96 pH] for red mullet (*Mullus barbatus*).

### 3.2. Antioxidant Potential of Collagen–Ginger Extract Systems

Antioxidant activity generally increased with increasing ginger concentration in the collagen–ginger systems, and significant differences were observed among several treatment groups (*p* < 0.05). The antioxidant activity of pure ginger extract was 86.88 ± 0.86%. Among the collagen–ginger combinations, the highest antioxidant activities were recorded in S3 (59.62 ± 2.02%), D3 (57.89 ± 1.44%), and M3 (55.04 ± 0.29%). A pronounced increase was observed from S1 to S2, whereas the increase from S2 to S3 was comparatively smaller. The control groups (Sc, Dc, and Mc) exhibited negligible antioxidant activity (Table 3).

### 3.3. Antimicrobial Activity of Collagen–Ginger Extract Systems

The antimicrobial activities of the collagen–ginger extract systems were determined using the agar well diffusion method, and the results were expressed as inhibition zone diameters (mm) (Figure 5). According to the findings, all samples exhibited antimicrobial activity against both bacterial strains. It was determined that the inhibition zones measured for *S. aureus* (7.15–9.65 mm) were higher compared to those for *E. coli* (6.15–8.70 mm). An increase in antimicrobial activity with increasing ginger extract content was observed in all groups (S1 < S2 < S3; D1 < D2 < D3; M1 < M2 < M3). The highest activity was obtained with pure ginger extract, whereas low inhibition zones (~6 mm) were detected in pure collagen samples. Statistical analysis revealed significant differences among the treatment groups (*p* < 0.05), confirming the positive effect of increasing ginger extract concentration on antimicrobial activity.

The cytocompatibility of the D3, M3, and S3 combinations was evaluated in human dermal fibroblast cells (HDFas) after 24 h of exposure using the MTT assay. Cell viability showed a concentration-dependent response, particularly at the higher combination ratios. Among the tested combinations, D3 exhibited relatively high cell viability across the concentration range, remaining close to or above 90% at most concentrations, although a significant decrease was observed at the highest tested ratios. In contrast, M3 caused the most pronounced reduction in cell viability, especially at 1.56%, 0.78%, 0.39%, and 0.20%, where viability decreased markedly compared with the negative control. The S3 combination also reduced cell viability at higher concentrations, but this effect was less severe than that observed with M3. At lower ratios, particularly 0.05%, 0.02%, and 0.01%, all combinations maintained cell viability levels comparable to the negative control, indicating improved cytocompatibility at these concentrations. Based on these findings, D3 at 0.20% and M3/S3 at 0.05% were selected as suitable non-cytotoxic or minimally cytotoxic concentrations for subsequent biosafety evaluations (Figure 6).

FDA/PI fluorescent staining was performed to further evaluate the cytocompatibility of the selected combination concentrations in HDFa cells after 24 h of exposure. In the negative control group, cells displayed predominantly green fluorescence, indicating preserved membrane integrity and high viability. Similarly, cells treated with D3, M3, and S3 showed extensive FDA-positive staining with only limited PI-positive cells, demonstrating that the selected concentrations did not induce prominent membrane damage or acute cytotoxicity. These fluorescence-based findings were consistent with the MTT results and supported the selection of D3 at 0.20%, M3 at 0.05%, and S3 at 0.05% as cytocompatible concentrations for subsequent biosafety analyses. The maintenance of viable cell morphology and the low level of dead-cell staining confirmed that the combinations exhibited acceptable short-term biocompatibility in human dermal fibroblast cells under the tested conditions (Figure 7).

Giemsa staining was performed to assess nuclear integrity and potential nuclear abnormalities in HDFa cells following 24 h exposure to the selected combination concentrations. The negative control group showed preserved cellular morphology with a low frequency of nuclear alterations, including micronuclei, lobbed nuclei, and notched nuclei. Similarly, cells treated with D3, M3, and S3 exhibited nuclear morphology comparable to the control group, without evidence of increased nuclear damage or abnormal chromatin organization. Quantitative scoring of 1000 cells per group showed that the mean nuclear abnormality frequency remained low in all treatment groups, with values of 0.020 ± 0.006 for D3, 0.019 ± 0.008 for M3, and 0.019 ± 0.003 for S3, compared with 0.017 ± 0.006 in the negative control. These findings indicate that the selected combination concentrations did not induce a meaningful increase in nuclear abnormalities in HDFa cells. The Giemsa-based nuclear integrity analysis therefore supports the MTT and FDA/PI results, confirming the biosafety and cytocompatibility of the selected concentrations under the tested conditions (Figure 8 and Table 4).

Annexin V-FITC/PI flow cytometry was used to define the apoptotic and necrotic cell death profile of HDFa cells after 24 h exposure to the selected combination concentrations. In this analysis, FL1−/FL2− cells were considered viable, FL1+/FL2− cells represented early apoptotic cells, FL1+/FL2+ cells represented late apoptotic/secondary necrotic cells, and FL1−/FL2+ cells represented necrotic cells. The negative control group showed a high viable cell population of 90.17%, with 5.78% early apoptotic, 2.89% late apoptotic/secondary necrotic, and 1.16% necrotic cells. Following D3 treatment, the viable population was 81.99%, while early apoptosis remained low at 2.84%. However, the late apoptotic/secondary necrotic population increased to 12.32%, and necrotic cells accounted for 2.84% of the total population. In the M3-treated group, 87.88% of cells remained viable, with 8.23% early apoptotic, 3.46% late apoptotic/secondary necrotic, and only 0.43% necrotic cells. Similarly, S3 treatment preserved a high viable cell fraction of 91.04%, with 2.99% early apoptotic, 4.10% late apoptotic/secondary necrotic, and 1.87% necrotic cells. When total apoptotic tendency was evaluated as the sum of early and late apoptotic/secondary necrotic populations, the values were 8.67% for the negative control, 15.16% for D3, 11.69% for M3, and 7.09% for S3. These data indicate that all selected combinations maintained the majority of HDFa cells in the viable population after 24 h exposure. Among the combinations, S3 showed the most comparable profile to the negative control, whereas D3 induced a moderate increase in the late apoptotic/secondary necrotic fraction. Nevertheless, the absence of a dominant necrotic population and the preservation of high viability percentages support the cytocompatibility of the selected concentrations, in agreement with the MTT, FDA/PI, and Giemsa findings (Figure 9).

## 4. Discussion

### 4.1. Collagen Characterization

#### 4.1.1. SDS-PAGE

In general, the electrophoretic patterns of collagen obtained using NADES were found to consist of two α chains (α1 and α2) and β chains (Figure 1). The obtained protein bands showed similarity with those reported for collagen extracted from Esox lucius [16] and *Dicentrarchus labrax* [17]. These findings indicate that the collagens obtained from the scales of gilthead seabream, European seabass, and red mullet exhibit Type I collagen characteristics. Ref. [18] reported that peptide hydrolysis patterns of acid-soluble collagens contain peptide fragments with a broad molecular weight distribution ranging approximately between 31–200 kDa. Similarly, ref. [19] reported that the protein patterns of collagens obtained by acid and pepsin methods are largely similar. However, it was determined that in collagen obtained by the pepsin method, the α1 chain was approximately 118.1 kDa and the α2 chain was approximately 107.4 kDa, which are lower than those of acid-extracted collagen, where the α1 and α2 chains were approximately 123.9 kDa and 112.7 kDa, respectively. The differences observed among studies in protein band patterns are thought to be associated with the hydrolytic effect of pepsin on telopeptide regions, as stated by [9].

#### 4.1.2. FTIR (Fourier Transform Infrared Spectroscopy)

FTIR spectra of collagens obtained from gilthead seabream, European seabass, and red mullet scales exhibited characteristic peaks corresponding to Amide A, Amide B, Amide I, Amide II, and Amide III bands. The Amide band values reported in other studies are presented in Table 5. The differences observed in the spectral bands are considered to arise from variations in the molecular structures of collagen [20]. When the Amide II band was examined, it was found that, compared to the normal absorption range reported in the literature (1550–1600 cm^−1^), the band position in this study was shifted to lower wavenumbers (1549–1547 cm^−1^ and 1546 cm^−1^). This shift is reported to be associated with the presence of hydrogen bonding in the collagen structure [20]. Ref. [18] reported that Amide A bands related to free N–H stretching vibrations appear at c. These values were found to be consistent with those reported by [21,22,23], as well as with the findings of the present study. Ref. [18] also reported that Amide I, Amide II, and Amide III bands are related to the degree of molecular order of collagen and play an important role in the formation of the triple-helix structure. When the FTIR spectra obtained in this study were compared with those reported in the literature, similar characteristic peaks were observed. The obtained results indicate that collagen extracted from fish scales using the NADES method largely preserves its natural triple-helical structure.

#### 4.1.3. SEM (Scanning Electron Microscopy)

The SEM images of collagen obtained from fish scales exhibited a smoother and more homogeneous structure compared to the typical collagen fibrillar architecture. In contrast, a fibrillar structure was observed in collagen obtained by acid and pepsin methods [19,25]. However, similar to our study, collagen morphologies reported by [26,27,28] were found to be comparable to the present findings. In addition, similar morphologies have also been observed in collagen obtained from Nile tilapia, Arothron stellatus, catla (*Catla catla*), rohu (*Labeo rohita*) and jellyfish skin [28,29].

#### 4.1.4. X-Ray Spectrum

The diffraction angles (2θ) were determined to be approximately 8.2° and 20.48° for red mullet, 8.2° and 22.36° for gilthead seabream, and 7.92° and 22.54° for European seabass. These two peaks are associated with the triple-helix structure of collagen [24]. These peaks were found to be consistent with those reported in other studies [19,20].

### 4.2. Antioxidant Potential of Collagen–Ginger-Based Combinations

When the antioxidant potential of collagen–ginger-based combinations was evaluated, the highest DPPH radical scavenging activity was obtained in the S3 group containing seabass collagen (59.62%), followed by the seabream (D3, 57.89%) and red mullet (M3, 55.04%) groups, respectively. In groups with lower ginger concentrations (S1, M1, and D1), antioxidant activity remained limited, and in particular, no measurable activity was detected in the S1 and M1 groups. The higher activity observed in pure ginger extract (86.88% inhibition) compared to all combinations indicates that the antioxidant activity is largely derived from phenolic compounds present in ginger extract [30]. However, this may be explained by the limited diffusion and interaction of these bioactive compounds within the collagen matrix. Indeed, the negative values observed in the control groups (Sc, Dc, and Mc) indicate that collagen alone does not possess DPPH radical scavenging capacity. The antioxidant activity of marine collagen peptides depends on amino acid composition, molecular weight, and peptide structure. In particular, amino acids such as Tyr, Met, His, Lys, and Trp contribute to radical scavenging activity, while the specificity of the protease used is also an important factor influencing the antioxidant properties of the resulting peptides [31]. The lower antioxidant activity values reported by [32] demonstrate that the source of collagen, extraction method, and molecular structure are determinant factors affecting this activity. In the literature, ref. [33] reported that seabass collagen exhibits significant DPPH activity at certain concentrations, while [34] demonstrated that collagen peptides show concentration-dependent increases in antioxidant capacity. These findings are consistent with the results obtained in the present study and support that antioxidant activity varies depending on both the structural properties of collagen and the concentration of bioactive compounds in the system. In the present study, antioxidant activity increased with rising ginger extract concentration; however, at higher concentrations, this increase tended to reach a plateau. The combination of collagen with ginger extract enhanced radical scavenging capacity, although lower activity values were obtained compared to the pure extract. This behavior may be explained by the limited diffusion and interaction of bioactive compounds within the collagen matrix. In the literature, it has been reported that the combined use of collagen and plant extracts can enhance biological activity and produce synergistic effects [35], which is consistent with the antioxidant activity enhancement observed upon ginger addition in this study.

### 4.3. Antimicrobial Activity of Collagen–Ginger-Based Combinations

Ginger (*Zingiber officinale*) belongs to the Zingiberaceae family, and its biological activity is mainly attributed to phenolic compounds and terpenoids, particularly gingerols, shogaols, and zingerone. The conversion of 6-gingerol into more active shogaols during thermal processing indicates that extraction and processing conditions play a decisive role in the bioactive compound profile and associated antimicrobial activity. Indeed, the antimicrobial effects of ginger vary depending on the type of extract; oleoresins have been reported to exhibit stronger effects with lower MIC values, whereas some essential oils are less effective, particularly against Gram-negative bacteria [36,37,38,39]. (In this study, fresh ginger was used, and the results demonstrated that ginger-derived bioactive compounds contributed directly to antimicrobial activity. It was observed that activity increased with increasing extract concentration, and that *Staphylococcus aureus* was more susceptible than Escherichia coli. This is attributed to the protective effect of the outer lipid membrane of Gram-negative bacteria, which is consistent with the literature [40,41].

As reported by [42], collagen is not considered to possess intrinsic antimicrobial activity. Therefore, the approximately 6 mm inhibition zone observed for the pure collagen samples was not considered evidence of intrinsic antimicrobial activity, as it corresponded to the diameter of the agar well. In contrast, the increase in inhibition zone diameter with increasing ginger extract concentration indicates that the antimicrobial activity primarily originated from ginger-derived bioactive compounds. These findings support the role of collagen as a potential carrier matrix for ginger-derived bioactive compounds rather than as an antimicrobial agent [40,41,43,44].

## 5. Conclusions

This study demonstrated the successful valorization of fish processing by-products as a sustainable source of collagen. Collagens extracted from the scales of gilthead seabream (*Sparus aurata*), European seabass (*Dicentrarchus labrax*) and red mullet (*Mullus barbatus*) exhibited preserved structural integrity and favorable physicochemical properties, confirming the suitability of fish scales as an alternative collagen source. The comprehensive characterization results highlighted the potential of these collagens for the development of functional biomaterial systems.

Furthermore, the incorporation of *Zingiber officinale* (ginger) extract into collagen matrices demonstrated the potential of the extracted collagens to serve as carrier matrices for natural bioactive compounds. The collagen–ginger combinations exhibited antioxidant and antimicrobial activities, with bioactivity generally increasing with ginger concentration. In addition, the favorable biocompatibility profiles of the combinations indicated their potential suitability for biomedical and functional applications.

Overall, the findings suggest that fish scale-derived collagens can serve not only as sustainable biomaterials but also as effective delivery matrices for plant-derived bioactive compounds. The present findings provide a preliminary scientific basis for the development of novel collagen-based functional materials and may contribute to future applications in food, pharmaceutical, and biomedical fields. Further studies focusing on encapsulation efficiency, controlled release behavior, and in vivo performance are recommended to fully elucidate the application potential of these systems.

## Figures and Tables

**Figure 1:**
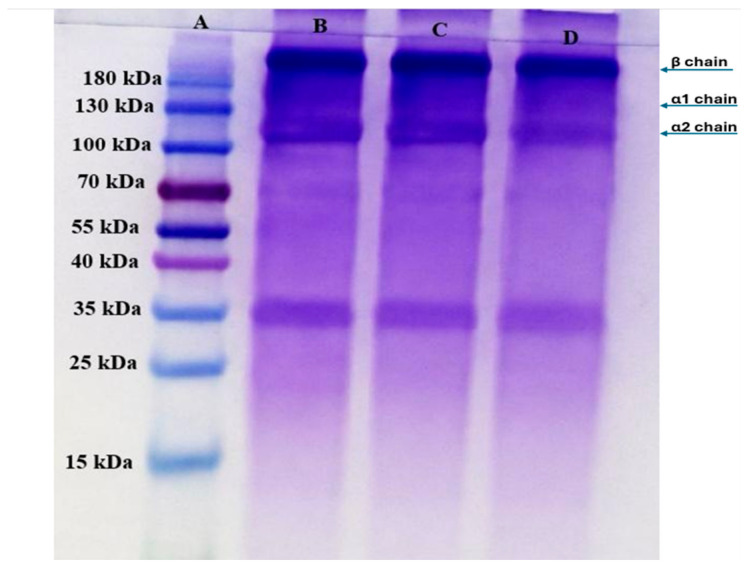
SDS-PAGE profile of collagen obtained from fish scales (**A**: molecular weight marker, **B**: Seabream, **C**: Seabass and **D**: Red mullet).

**Figure 2:**
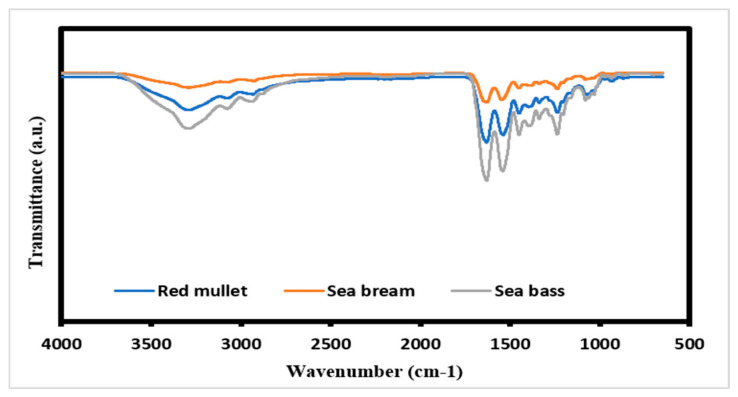
FTIR analysis of collagen obtained from fish scales.

**Figure 3:**
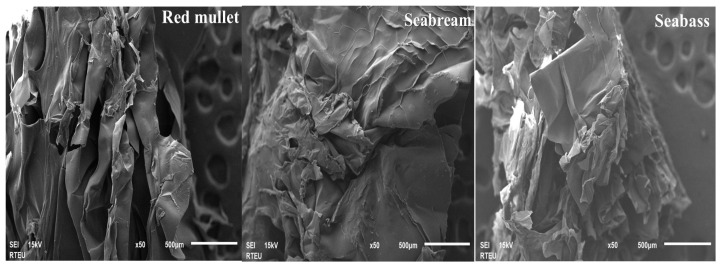
SEM image of lyophilized collagen obtained from fish scales.

**Figure 4:**
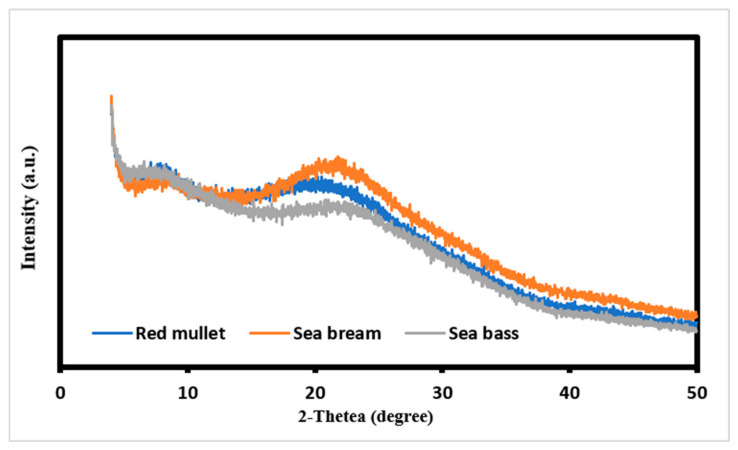
X-ray spectrum of collagen obtained from fish scales.

**Figure 5:**
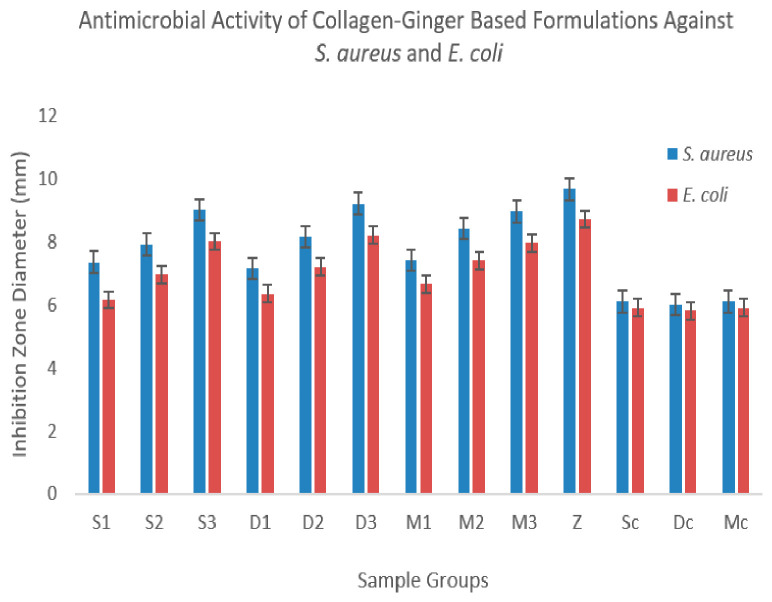
The inhibition zone diameters (mm) produced by collagen–ginger-based combinations on *Staphylococcus aureus* and *Escherichia coli* in different sample groups (S1–S3, D1–D3, M1–M3, Z, Sc, Dc, Mc) are shown. Values are presented as mean ± standard deviation.

**Figure 6:**
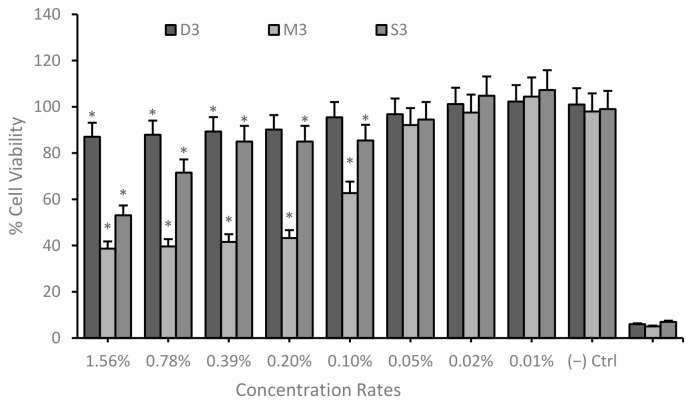
MTT-based cell viability assessment of D3, M3, and S3 combinations in human dermal fibroblast cells (HDFas) following 24 h exposure. Cells were treated with serial combination ratios ranging from 1.56% to 0.01%, and untreated cells were used as the negative control. Data are presented as mean ± SD. Asterisks indicate statistically significant differences compared with the negative control (* *p* < 0.05).

**Figure 7:**
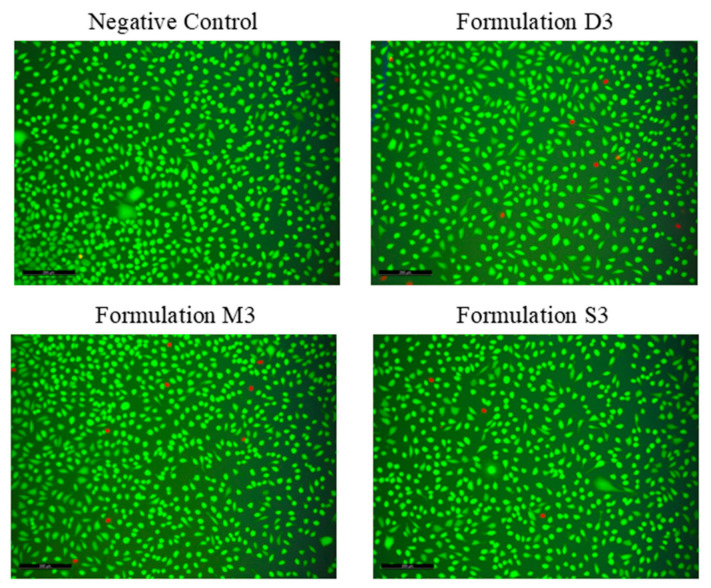
FDA/PI fluorescence staining of human dermal fibroblast cells (HDFas) following 24 h exposure to selected concentrations of D3, M3, and S3 combinations. The tested concentrations were D3: 0.20%, M3: 0.05%, and S3: 0.05%. FDA-positive viable cells are shown in green, whereas PI-positive non-viable cells are shown in red. Scale bar: 200 µm.

**Figure 8:**
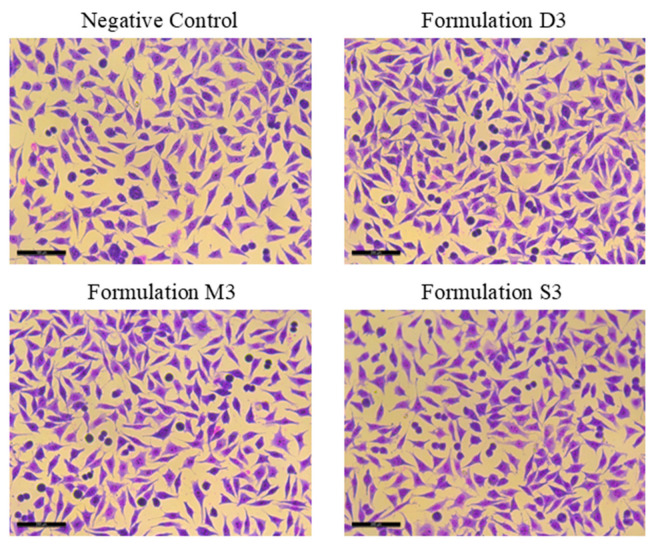
Giemsa nuclear integrity analyses of combinations on the human dermal fibroblast (HDFa) cell line for 24 h of application (D3: 0.20%, M3: 0.05% and S3: 0.05%, Scale Bar: 200 µm).

**Figure 9:**
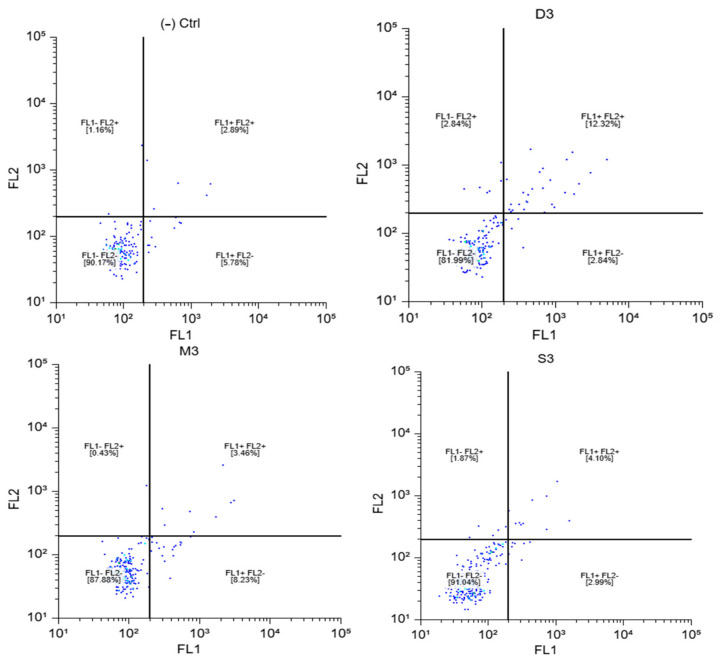
Flow cytometry cell death mechanism analyses of combinations on the human dermal fibroblast (HDFa) cell line for 24 h of application (D3: 0.20%, M3: 0.05% and S3: 0.05%).

**Table 1:** Composition of collagen–ginger combinations prepared from different fish species.

Group	Fish Species	Collagen (4% Stock Solution)	Ginger Extract (10% Stock Solution)	Mixing Ratio (%, *v*/*v*)
M_c_	Red mullet	100	-	-
S_c_	Seabass	100	-	-
D_c_	Seabream	100	-	-
M_1_	Red mullet	4	1	4:1
S_2_	Red mullet	3	2	3:2
D_3_	Red mullet	2	3	2:3
M_1_	Seabass	4	1	4:1
S_2_	Seabass	3	2	3:2
D_3_	Seabass	2	3	2:3
M_1_	Seabream	4	1	4:1
S_2_	Seabream	3	2	3:2
D_3_	Seabream	2	3	2:3

**Table 2:** Evaluation of FTIR peak areas of the obtained collagen structures.

Wave Number (cm^−1^)	Band	Assignment	Sea Bream	Red Mullet	Sea Bass
3300–3440 cm^−1^	Amide A	N–H stretching vibration and hydrogen bonding	3290	3365	3298
2920–2935 cm^−1^	Amide B	Asymmetric CH_2_ stretching vibration	2929	2931	2932
1600–1690 cm^−1^	Amide I	C=O stretching vibration	1633	1634	1634
1480–1575 cm^−1^	Amide II	N–H bending and C–N stretching vibrations	1549	1547	1546
1220–1300 cm^−1^	Amide III	C–N stretching and N–H deformation vibrations	1238	1237	1242

**Table 3:** DPPH radical scavenging activity of collagen–ginger-based combinations (% inhibition, mean ± SD, *n* = 3). Values with different superscript letters (a–e) are significantly different (*p* < 0.05).

Control		Seabass (S)		Seabream (D)		Red Mullet (M)	
S_c_	−1.58 ± 0.79 ^de^	S_1_	0.00 ± 0.00 ^de^	D_1_	3.36 ± 0.86 ^d^	M_1_	0.00 ± 0.00 ^de^
D_c_	−2.14 ± 0.14 ^de^	S_2_	48.22 ± 0.72 ^c^	D_2_	48.37 ± 0.21 ^c^	M_2_	47.10 ± 0.72 ^c^
M_c_	−3.56 ± 0.43 ^e^	S_3_	59.62 ± 2.02 ^a^	D_3_	57.89 ± 1.44 ^a^	M_3_	55.04 ± 0.29 ^b^

**Table 4:** Quantitative assessment of nuclear abnormalities in human dermal fibroblast cells (HDFas) following 24 h exposure to selected concentrations of D3, M3, and S3 combinations. Micronuclei (MN), lobbed nuclei, and notched nuclei were scored in 1000 cells per group, and nuclear abnormality frequency was expressed as mean NA/1000 cells ± SD. Superscript letters indicate statistical comparison between groups; groups sharing the same letter are not significantly different from each other (*p* > 0.05).

Groups	Total MN	Total Lobbed	Total Notched	Mean NA/1000 Cells ± SD
(−) Control	6	4	7	0.017 ± 0.006 ^a^
D3	7	5	8	0.020 ± 0.006 ^a^
M3	6	7	6	0.019 ± 0.008 ^a^
S3	5	7	7	0.019 ± 0.003 ^a^

**Table 5:** FTIR amide band values reported in other studies.

References	Amide A	Amide B	Amide 1	Amide 2	Amide 3
[19]	3276.4	2931.4	1628.6	1541.2	1235.6
[20]	3431.7	2931.2	1643	1546.6	1448.2
[16]	3312	3076	1653	1548	1235
[24]	3314.5	2936	1658.7	1543.4	1232.7

## Data Availability

The datasets generated during the current study are available from the corresponding author on request.
